# ^18^F-FDG Uptake on PET/CT in Symptomatic versus Asymptomatic Carotid Disease: a Meta-Analysis

**DOI:** 10.1016/j.ejvs.2018.03.028

**Published:** 2018-08

**Authors:** Mohammed M. Chowdhury, Jason M. Tarkin, Nicholas R. Evans, Elizabeth Le, Elizabeth A. Warburton, Paul D. Hayes, James H.F. Rudd, Patrick A. Coughlin

**Affiliations:** a Division of Vascular and Endovascular Surgery, Addenbrooke's Hospital, Cambridge University Hospital Trust, Cambridge, UK; b Division of Cardiovascular Medicine, University of Cambridge, Addenbrooke's Hospital, Cambridge, UK; c Division of Clinical Neurosciences, University of Cambridge, Addenbrooke's Hospital, Cambridge, UK

**Keywords:** Inflammation, Microcalcification, Peripheral arterial disease, Positron emission tomography

## Abstract

**Introduction:**

The role of positron emission tomography (PET)/computed tomography (CT) in the determination of inflammation in arterial disease is not well defined. This can provide information about arterial wall inflammation in atherosclerotic disease, and may give insight into plaque stability. The aim of this review was to perform a meta-analysis of PET/CT with ^18^F-FDG (fluorodeoxyglucose) uptake in symptomatic and asymptomatic carotid artery disease.

**Methods:**

This was a systematic review, following PRISMA guidelines, which interrogated the MEDLINE database from January 2001 to May 2017. The search combined the terms, “inflammation”, “FDG”, and “stroke”. The search criteria included all types of studies, with a primary outcome of the degree of arterial vascular inflammation determined by ^18^F-FDG uptake. Analysis involved an inverse weighted variance estimate of pooled data, using a random effects model.

**Results:**

A total of 14 articles (539 patients) were included in the meta-analysis. Comparing carotid artery ^18^F-FDG uptake in symptomatic versus asymptomatic disease yielded a standard mean difference of 0.94 (95% CI 0.58–1.130; *p* < .0001; *I*^2^ = 65%).

**Conclusions:**

PET/CT using ^18^F-FDG can demonstrate carotid plaque inflammation, and is a marker of symptomatic disease. Further studies are required to understand the clinical implication of PET/CT as a risk prediction tool.

**What this paper adds**

This meta-analysis, including 14 studies, compared carotid artery uptake in symptomatic versus asymptomatic disease, and demonstrated a significantly higher tracer uptake in symptomatic disease. Although PET/CT imaging in atheroma is a research tool and is currently limited in its clinical applicability, it may provide information about plaque biology, and therefore in the future, risk of stroke in carotid disease patients.

## Introduction

Atherosclerosis related cardiovascular disease is a major global health problem, and is the leading cause of death in every region of the world.^1^ Alongside myocardial infarction, stroke is a major cause of mortality and morbidity. Approximately 80% of strokes are ischaemic in nature, with the carotid artery deemed the embolic source in 10%. There is strong evidence that early carotid endarterectomy is key in reducing stroke rates in patients with significant symptomatic ipsilateral carotid disease,^2^ whereas asymptomatic disease is increasingly managed by conservative treatment (best medical therapy).^3^ Only a small proportion of asymptomatic patients with a significant carotid artery stenosis will develop a stroke or transient ischaemic attack (TIA) and as such the key is to identify those patients with an “at risk” plaque who could then be offered carotid endarterectomy.^4^

Atherosclerotic plaques, formed because of a build up of lipids and inflammatory cells within the arterial wall, often occur in areas of low sheer stress, including arterial bifurcations, as the disturbed blood flow increases the expression of cellular transcription factors and adhesion molecules responsible for recruiting circulating monocytes.^5^, ^6^ High risk plaque characteristics include a thin fibrous cap, inflammatory cell infiltration, large lipid core, paucity of smooth muscle cells, and microcalcification. Mechanisms determining transition of high risk plaques to either a more stable phenotype or to plaque rupture are incompletely understood; however, inflammation is increasingly recognised as a precipitant for plaque rupture, which within the carotid circulation results in symptomatic embolic consequences, namely transient ischaemic attacks or stroke.^7^

Positron emission tomography (PET) is a nuclear imaging technique with a high sensitivity, meaning that it can detect picomolar concentrations of a tracer (a radiolabelled ligand) of interest. As the tracer decays, an annihilation reaction produces photons. The release of gamma radiation is detected by the PET scanner and used to create a tomographic map of tracer distribution within the body. A common measure of tracer uptake used to determine tracer activity is the standardised uptake value (SUV). When SUV is corrected for blood pool activity (the circulating level of tracer in the venous system), this is termed the tissue to background ratio (TBR).

Fluorodeoxyglucose (^18^F-FDG) is a radiolabelled glucose analogue, which is taken up by all glucose metabolising cells. Specifically, FDG competes with endogenous glucose for facilitated transport sites (GLUT-1 and GLUT-3). After phosphorylation FDG becomes trapped within the cells it has entered as it lacks the necessary 2′ hydroxyl group needed to advance in the glycolytic pathway. Macrophages are active in the atherosclerotic plaque and can metabolise free fatty acids, but in the plaque, they prefer glucose: activated macrophages have an increased expression of GLUT-1 and -3 receptors. FDG uptake is therefore a function of macrophage density and activation, and therefore plaque activity.^8^

The evidence base for PET/CT imaging in arterial disease is continually developing in a number of vascular beds. The ease of imaging the carotid artery using a number of techniques, the availability of atherosclerotic plaques for histological interrogation as a result of carotid endarterectomy and the devastating consequence of stroke make the carotid artery a pertinent one to study. As such, this meta-analysis focuses on how, specifically ^18^F-FDG PET/CT imaging can be applied to carotid artery atherosclerosis.

## Methods

This study followed the Preferred Reporting Items for Systematic Reviews (PRISMA) checklist.^9^ Ethics committee approval was not required as the study was a systematic review/meta-analysis. The study is a meta-analysis evaluating the role of ^18^F-FDG PET/CT imaging in patients with carotid artery atherosclerotic disease. The primary outcome was to determine differences in ^18^F-FDG tracer uptake between significant symptomatic and asymptomatic carotid stenosis.

### Search strategies

An electronic search was undertaken using EMBASE and PUBMED to search the MEDLINE database from January 2001 to May 2017. The search combined the terms, “inflammation”, “FDG”, and “stroke”.

### Search criteria

Inclusion criteria comprised all study types including randomised controlled, cohort, case–control, case series, and experimental studies with human subjects undergoing PET/CT scans examining carotid disease in at least five patients. Studies were excluded if they, included coronary or aortic analysis of PET/CT imaging, involved imaging within animals, studies of vasculitis, or if the study did not provide clinical data (symptomatology). Furthermore, studies were also excluded if they examined carotid uptake in the absence of carotid atherosclerosis.

### Data extraction and synthesis

Data were extracted by one researcher (M.M.C.) and checked by another researcher (J.M.T.) using a standardised data capture form developed prior to the onset of the study. Disagreements were resolved by re-extraction or by third party adjudication (P.A.C.) when necessary.

Collected study characteristics included number of subjects undergoing PET/CT and number of centres involved in the study. Furthermore, patient population, dose of FDG injected, uptake time, imaging protocols, primary endpoint measures for PET/CT, and the main findings were all collected and tabulated, as per published guidance.^10^

### Statistical analysis

Estimation of the global effect for the primary outcome for carotid disease (^18^F-FDG uptake in symptomatic versus asymptomatic plaques) was assessed through an inverse variance weighted estimate of the pooled data (where applicable), using the random effects model. Given the continuous nature of data (SUV_max_/TBR_max_) application of the Mantel–Haenszel method was used. The standardised mean difference was used given the variability in reporting outcomes (SUV and TBR). Analysis was performed using Review Manager Analysis software (RevMan 5.3.5, Nordic Cochrane Centre, Copenhagen, Denmark).

## Results

### Included studies

**Figure 1:**
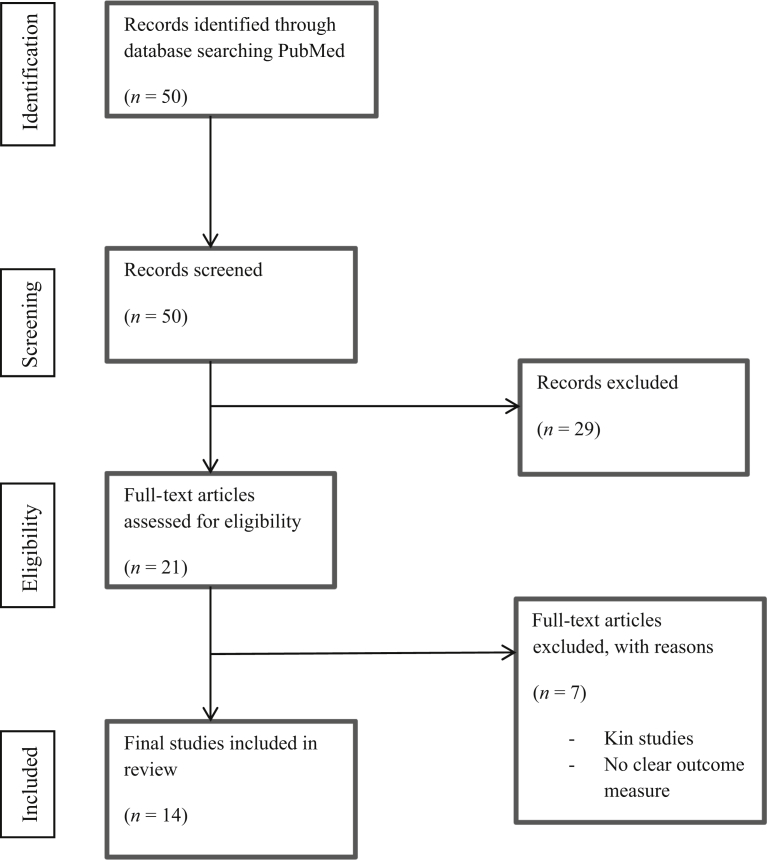
Flow diagram of systematic review synthesis.

A total of 50 titles were initially identified from the search and their titles and abstracts were screened (Fig. 1). Of those, 29 were excluded as they failed to meet the inclusion criteria. The remaining 21 were assessed for eligibility via full text review. Two studies were then excluded because of being (earlier) replicated studies and a further study excluded because of inadequate reporting of objective outcome data, and have not been presented in the citation list. Four articles did not have a direct comparison of symptomatic versus asymptomatic disease. The remaining 14 articles were included in the meta-analysis and form the study population analysed (PRISMA Flow Chart; Fig. 1). The total number of patients identified from the included studies was 539 (268 symptomatic, 271 asymptomatic). The mean ^18^F-FDG injected dose was 306.3 ± 91.4 MBq.

### ^18^F-FDG tracer uptake in symptomatic and asymptomatic carotid disease

**Figure 2:**
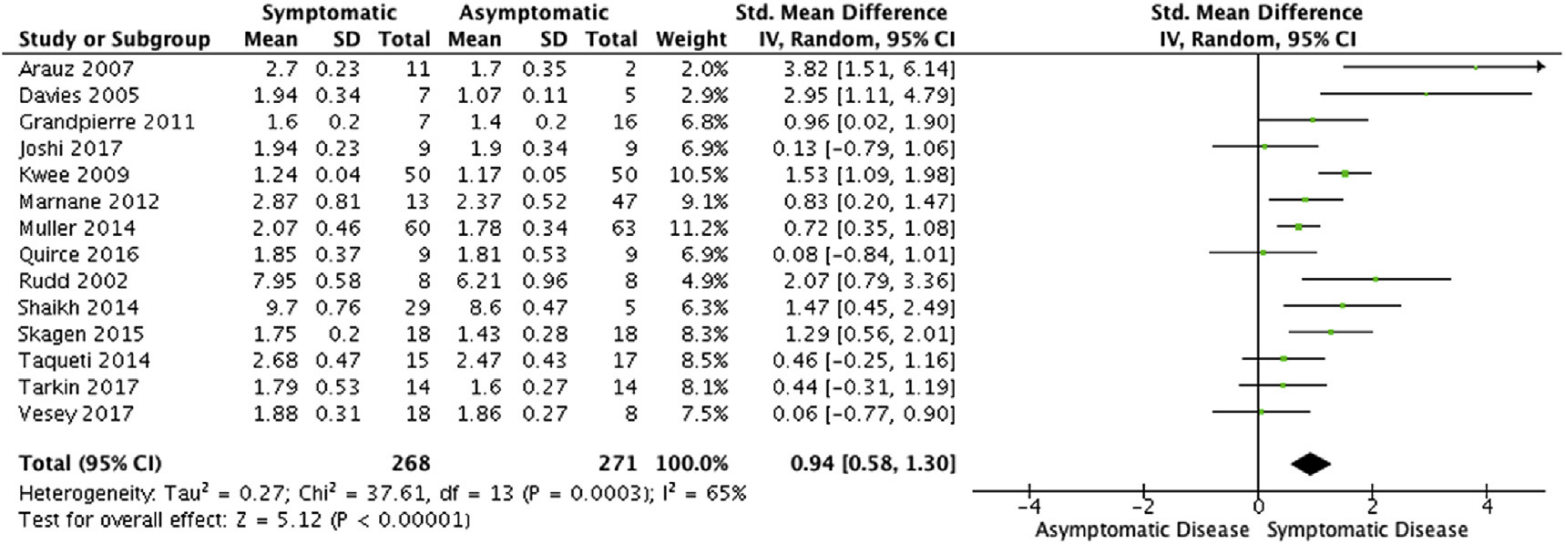
Forest plot for analysis of 18F-FDG uptake in symptomatic versus asymptomatic carotid disease (*p* < .00001).

A total of 539 patients were included within this analysis (mean age 71.2 ± 3.5, 76% male). Pooled comparisons of studies that analysed a difference in symptomatic versus asymptomatic carotid atherosclerotic disease demonstrated that ^18^F-FDG tracer uptake was significantly higher in symptomatic carotid lesions (standard mean difference 0.94; 95% CI 0.58–1.30; *p* < .0001; I^2^ = 65%, Fig. 2). A summary of study details is shown in Table 1.^11^, ^12^, ^13^, ^14^, ^15^, ^16^, ^17^, ^18^, ^19^, ^20^, ^21^, ^22^, ^23^, ^24^

**Table 1:** Included study characteristics.

Study and design	Population, age, average time from symptoms to scan	Dose FGD injected	Uptake time and protocol	Primary endpoint measure	Findings (FDG)
Rudd et al. 2002^11^ Patient cohort study, IV administration of ^18^F-FDG with carotid disease, PET/CT, histology	*n* = 8 patients, symptomatic disease (mean age 63.5, 75% male, 3.5 months)	370 MBq over 60 s	190 min ± 6 Reconstructed images with 3D re-projection, corrected for attenuation	FDG accumulation rate, volumes of interest (VOI)	Higher ^18^F-FDG PET/CT signals in symptomatic versus asymptomatic disease (*p* = .005) verified histologically (CD68)
Davies et al. 2005^12^ Prospective cohort study, IV administration of ^18^F-FDG with carotid disease, PET, MRI	*n* = 12 patients, culprit carotid disease (mean age 71, 83% male, 64 days)	185 MBq	120 min Reconstructed emission images using PROMIS 3D filtered back projection algorithm	Uptake ratio (FDG in plaque divided by normal arterial wall FDG)	Higher FDG uptake ratios in symptomatic carotid disease
Arauz et al. 2007^13^ Prospective cohort study, IV administration of ^18^F-FDG with carotid disease, PET/CT	*n* = 13 patients, high and low uptake (mean age 66.1, 69% male, 25 days)	370 MBq	90 min 6 h fast, image acquisition in 3D mode, attenuated corrected	Standardised uptake values (SUVs)	Patients with symptomatic carotid disease had higher FDG uptake, as well as with stenosis (*p* ≤ .001)
Kwee et al. 2011^14^ Prospective cohort study, IV administration of ^18^F-FDG with carotid disease, PET/CT	n = 50 patients, symptomatic carotid disease (mean age 67.8, 68% male, 33.3 days)	200 MBq	60 min 12 h fast, 3D mode, 120 kVp, 175 mAs, field of view 250 × 250 mm.	Standardised uptake values (SUVs)	Significant correlation between FDG signal and CT characteristics in symptomatic plaque (*p* < .05)
Grandpierre et al. 2011^15^ Retrospective, cohort study, IV administration of FDG, PET/CT	*n* = 23 patient, cancer patients admitted with stroke due to carotid disease (mean age 66, 86% male, 7 months)	400 MBq	60 min 6 h fast, 3D mode, 120 kV, 80 mAs, field of view 512 × 512 mm, 3 min per bed position	Standardised uptake values (SUVs)	Higher FDG uptake in patient who had stroke in the carotid artery was compared to no stroke patients (*p* = .006)
Marnane et al. 2012^16^ Patient cohort study, symptomatic stroke patients, IV administration of ^18^F-FDG, PET/CT	*n* = 60 patients 13 recurrences (mean age 67.3, 85% male, 5 days) 47 no recurrence (mean age 71.6, 68% male, 7 days)	320 MBq	120 min 6 h fast, image acquired in 3D mode, 2 bed positions for 10 min	Standardised uptake values (SUVs)	Higher uptake of ^18^F-FDG in patients with recurrent strokes (*p* = .02), mean plaque FDG only predictor of stroke recurrence (HR 6.1, 1.3–28.8, *p* = .02)
Muller et al. 2014^17^ Patient cohort study, symptomatic stroke patients, IV administration of ^18^F-FDG, PET/CT	*n* = 123 60 symptomatic 63 asymptomatic (mean age 72, 76% male)	370 MBq	90 min 6 h fast, 3D mode, matric 336 × 336 pixels, attenuated corrected	Target to background ratios (TBRs)	Significantly higher FDG uptake in symptomatic high risk carotid plaques (*p* < .0018)
Taqueti et al. 2014^18^ Patient cohort study, IV administration of ^18^F-FDG, PET/CT, DCE-MRI	*n* = 32 patients symptomatic disease (mean age 68, 50% male)	370 MBq	90 min 3D list mode, in 1 bed position over 20 min Radiation exposure 8 mSv	Target to background ratios (TBRs)	^18^F-FDG signals correlate highly with markers of macrophage density, in symptomatic plaque (*p* < .001)
Shaikh et al. 2014^19^ Patient cohort study, IV administration of ^18^F-FDG, cf. femoral plaque	*n* = 35 patients 29 symptomatic carotid and 5 asymptomatic (mean age 67, 76% male)	185 MBq	60 min 6 h fast, 3D mode, 120 kV attenuation correc. Radiation 27.4 mSv	Regions of interest (ROI) and hot average	Significantly higher hot average in symptomatic vs. asymptomatic carotid plaques (*p* = .053)
Skagen et al. 2015^20^ Prospective patient cohort study, IV administration of ^18^F-FDG, PET/CT	*n* = 36 patients 18 symptomatic, 18 asymptomatic (mean age 67, 72% male)	370 MBq	90 min 6 h fast, 2D OSEM algorithm, with matrix 256 × 256 pixels	Standardised uptake values (SUVs)	Significantly higher hot average in symptomatic versus asymptomatic carotid plaques (*p* = .003)
Quirce et al. 2016^21^ Prospective patient cohort study, IV administration of ^18^F-FDG and ^18^F—NaF, PET/CT	*n* = 18 patients 9 symptomatic, 9 asymptomatic (mean age 67, 88% male)	450 MBq	180 min 6 h fast, 5 min per bed space, 130 kV, 50 mAs 11.3 mSv radiation dose	Standardised uptake values (SUVs)	No significant difference in FDG uptake in symptomatic versus asymptomatic plaque (*p* = .85)
Vesey et al. 2017^22^ Prospective case control study, IV administration of ^18^F-FDG and NaF, high risk plaque	*n* = 26 patients 18 carotid group (mean age 71.7, 66.7% male) 8 control group (mean age 66.1, 50% male)	200 MBq	90 min 6 h fast, 3D mode, 120 kV, 2 bed positions for 20 min per bed.	Target to background ratios (TBRs)	^18^F-FDG higher uptake in culprit vessel vs. control (*p* = .014), log10 transformed data
Tarkin et al. 2017^23^ Prospective cohort study, IV administration of ^18^F-FDG, PET/CT, 68Ga-DOTATATE	*n* = 28 patients 14 in carotid group (mean age 71, 75% male, 18 days) 14 in control (mean age 72.3, 76% male)	248.1 ± 22.3 MBq	90 min 6 h fast, 4 bed positions for 15 min, radiation 30 mSv, VUE FX recons	Target to background ratios (TBRs)	Significant uptake of FDG in symptomatic plaques versus control (*p* < .001)
Joshi et al. 2017^24^ Prospective cohort study, IV administration of ^18^F-FDG, PET/CT, FMISO tracer	*n* = 16 patients 8 patients in carotid group (mean age 73, 70% male, 16 days) 8 patients control (mean age 71, 50% male)	250 MBq	120 min 6 h fast, one bed position for 15 min, 3D mode, VUE FX recons, radiation 10.4 mSv	Target to background ratios (TBRs)	Higher uptake of FDG in culprit lesions (TBR 1.94 versus 1.90), despite *p* > .05 trend toward significance

## Discussion

This study is the first to systematically review and evaluate the role of ^18^F-FDG imaging of inflammation within carotid artery atherosclerotic disease. The findings demonstrate that ^18^F-FDG accurately differentiates between symptomatic and asymptomatic carotid plaques and is validated to accurately image areas of high inflammation within the symptomatic carotid atherosclerotic plaque, validated by histological analysis. Rudd et al.^11^ performed the first prospective validation study using ^18^F-FDG PET/CT to detect and quantify vascular inflammation. They observed significantly higher FDG tracer uptake within symptomatic carotid plaques than the contralateral asymptomatic carotid artery. Further studies have continued to explore the role of PET/CT imaging within the carotid plaque and the results form this meta-analysis.

Currently, the indication for carotid intervention is an ipsilateral neurovascular event with a >50% internal carotid artery stenosis (NASCET criteria, ESC Guidelines^25^). Even in patients with stenosis >70%, the number needed to treat (NNT; defined as the number of patients who would have to undergo carotid endarterectomy in order to prevent one long-term adverse event) is six for patients with 70–99% stenosis and 15 for patients with 50–69% stenosis.^26^ As such, significant numbers of patients undergo potentially needless and high risk surgery. PET/CT imaging could play a role in specific risk stratification for these patients, aiding clear identification of an active carotid plaque comparison with patients who may have non-carotid causes of symptoms.

More debatable is the role of intervention in patients with a significant yet asymptomatic carotid stenosis. Data from randomised trials shows that surgery confers an absolute risk reduction in stroke of approximately 5%, equating to a NNT of approximately one in 40. There are a number of clinical and imaging features that may identify those patients with a higher rate of stroke despite optimal medical therapy yet because of the small event rate, large scale screening studies will be needed to prove validity which may limit the role of PET/CT imaging in part due to radiation exposure. This can be overcome with PET/magnetic resonance imaging.^27^ PET imaging provides the advanced molecular imaging, with high sensitivities, that mainstream vascular imaging modalities do not offer.^28^ The heterogeneity of the data included in this analysis (as discussed below) means that it is difficult to ascertain the cut off value for ^18^F-FDG uptake (TBR/SUV) that defines a “high risk plaque”. Many of the reported studies do not report individual patient ^18^F-FDG uptake values, rendering interpretation of the role of PET/CT imaging in asymptomatic disease ambiguous at best. While the larger longitudinal studies studying arterial inflammation have tended to focus on non-atherosclerotic oncological populations, this means that there is still a need for a large longitudinal prospective study of asymptomatic patients to assess the truly predictive role of ^18^F-FDG PET imaging in progression of carotid disease activity.^29^

Pharmacotherapy trials have demonstrated both positive and negative effects on signal uptake in carotid atheroma,^30^ yet there are further successful trials that have used PET/CT as an imaging biomarker in other arterial beds, for example the aorta and iliac vessels,^31^ and it is likely to be that such imaging has distinct advantages as an outcome measure.

It is recognised that this field of research is still in its infancy and understanding the role of molecular imaging in atherosclerotic risk stratification is continuing. Owing to a lack of large adequately powered studies, the formal assessment was varied and generally poor due to insufficient data. Furthermore, another limitation to the included data is the lack of reporting the time relationship between imaging and symptom onset. This was only presented in eight out of the 14 studies, and requires clarification with further molecular carotid imaging studies.

Of note, one ever evolving aspect in PET research methodology is the optimisation of imaging protocols with respect to the assessment of atheroma (variation across included studies; Table 1). Publication of a recent position paper^32^ by the European Association of Nuclear Medicine on PET imaging in atherosclerosis has led to the creation of technical recommendations when undertaking ^18^F-FDG PET. These include an injected activity of between 3 and 4 MBq/kg body weight, acquisition of PET images 120 min post injection, and an ideal blood glucose level of less than 7.0**–**7.2 mmol/L. It is evident that given this, more recent studies are using these recommendations within their protocol development and methodology.

The development of a number of novel tracers which identify other pathological processes (hypoxia, neo-angiogenesis^24^) alongside more specific markers of inflammation (^68^Ga-DOTATATE)^23^ will continue to enhance the role of molecular imaging. Specifically, there is now high quality evidence regarding the role of the tracer ^18^F—NaF (sodium fluoride; a marker of microcalcification) in risk stratifying atherosclerotic plaques within the coronary circulation which is timely given the common issues of tracer overspill within the myocardium which limits the role of ^18^F-FDG within the coronary circulation.^33^ Similar data are now available for ^18^F-FDG within the carotid circulation where ^18^F-FDG molecular imaging can highlight culprit high risk carotid plaques, while also correlating strongly with predicted cardiovascular risk.^22^

## Conclusion

For over a decade, ^18^F-FDG PET/CT imaging in the carotid circulation has provided an enhanced ability to study atherosclerotic inflammation in symptomatic patients. The studies presented here demonstrate that it is well validated, reproducible, and may have the ability to differentiate vulnerable plaque (in symptomatic patients) from more stable plaque (in asymptomatic patients). ^18^F-FDG uptake is significantly higher in symptomatic carotid disease than in asymptomatic disease. Research methodologies are being refined, and the role of PET/CT imaging in atheroma has the reality of becoming a risk stratification tool. However, much work is still required before its use in clinical practice. Current data are limited predominantly to the carotid and coronary circulation and as such focus needs to turn to other vascular beds and specifically the lower limb arterial tree.

## Funding

M.M.C is supported by the Royal College of Surgeons of England Fellowship Programme (Freemasons' Award) and a British Heart Foundation Research Fellowship award (FS/16/29/31957). J.M.T is supported by a Wellcome Trust Research Training Fellowship (104492/Z/14/Z). N.R.E is supported by a research fellowship from the Dunhill Medical Trust (RTF44/0114). J.H.F.R is part-supported by the NIHR Cambridge Biomedical Research Centre, the British Heart Foundation, HEFCE and the Wellcome Trust. P.A.C is supported by the Circulation Foundation, British Heart Foundation and the Dunhill Medical Trust.

## Conflict of Interest

None.
