# Supplementary material for: 18F-FDG Uptake on PET/CT in Symptomatic versus Asymptomatic Carotid Disease: a Meta-Analysis
Source: Eur J Vasc Endovasc Surg. 2018 Aug;56(2):172–9. doi: 10.1016/j.ejvs.2018.03.028 (PMC6105570; doi:10.1016/j.ejvs.2018.03.028)
Supplement: Multimedia component 1 [file mmc1.docx]

**Supplementary data - Excluded studies:**

| Author | Title | Reason for exclusion |
| --- | --- | --- |
| Croteau et al, 2003 | Quantitative gated PET for the assessment of left ventricular function in small animals. | Animal study |
| Tawakol et al, 2006 | In vivo 18F-fluorodeoxyglucose positron emission tomography imaging provides a non-invasive measure of carotid plaque inflammation in patients. | Only a symptomatic cohort analysed |
| Tang et al, 2008 | Combined PET-FDG and USPIO-enhanced MR imaging in patients with symptomatic moderate carotid artery stenosis. | MRI analysis |
| Krupinski et al, 2008 | Angiogenesis and inflammation in carotid atherosclerosis. | Review of data |
| Izquierdo-Garcia et al, 2009 | Comparison of methods for magnetic resonance-guided [18-F]fluorodeoxyglucose positron emission tomography in human carotid arteries: reproducibility, partial volume correction, and correlation between methods. | MRI analysis |
| Potter et al, 2009 | Effect of long-term homocysteine reduction with B vitamins on arterial wall inflammation assessed by fluorodeoxyglucose positron emission tomography: a randomised double-blind, placebo-controlled trial. | Effect of intervention on uptake (vitamins) |
| Schroeter et al, 2009 | Neuroinflammation extends brain tissue at risk to vital peri-infarct tissue: a double tracer [11C]PK11195- and [18F]FDG-PET study. | Animal study |
| Kato et al, 2009 | Evaluation and comparison of 11C-choline uptake and calcification in aortic and common carotid arterial walls with combined PET/CT. | No FDG uptake analysis |
| Rominger et al, 2009 | 18F-FDG PET/CT identifies patients at risk for future vascular events in an otherwise asymptomatic cohort with neoplastic disease. | Neoplastic disease in asymptomatic cohort |
| Hermus et al, 2010 | Advanced carotid plaque imaging. | Review of current literature |
| Graebe et al, 2010 | 18FDG PET and ultrasound echolucency in carotid artery plaques. | No comparison with asymptomatic cohort – emphasis on US |
| Chen et al, 2010 | (18)F-fluorodeoxyglucose PET imaging of coronary atherosclerosis and plaque inflammation. | Review of current literature |
| Moustafa et al, 2010 | Carotid plaque inflammation is associated with cerebral microembolism in patients with recent transient ischemic attack or stroke: a pilot study. | MRI analysis |
| Fukumoto et al, 2011 | Multiparametric assessment of acute and subacute ischemic neuronal damage: a small animal positron emission tomography study with rat photochemically induced thrombosis model. | Animal study |
| Beer et al, 2012 | Systemic arterial inflammation, measured with 18FDG-PET, is common amongst subjects with both recent and prior cerebrovascular disease. | No CT co-registration |
| Hermann et al, 2012 | Non-FDG imaging of atherosclerosis: will imaging of MMPs assess plaque vulnerability? | Review of current literature |
| Yu et al, 2012 | Aortic vascular inflammation in psoriasis is associated with HDL particle size and concentration: a pilot study. | No carotid analysis, no stroke subgroup |
| Chen et al, 2013 | Targeted PET/CT imaging of vulnerable atherosclerotic plaques: microcalcification with sodium fluoride and inflammation with fluorodeoxyglucose. | Review of current literature |
| Saito et al, 2013 | Validity of dual MRI and F-FDG PET imaging in predicting vulnerable and inflamed carotid plaque. | MRI analysis |
| Subramanian et al, 2013 | High-dose atorvastatin reduces periodontal inflammation: a novel pleiotropic effect of statins. | Effect of intervention on uptake (statin) |
| Truijman et al, 2013 | Combined 18F-FDG PET-CT and DCE-MRI to assess inflammation and microvascularization in atherosclerotic plaques. | No asymptomatic comparison and MRI analysis |
| Figueroa et al, 2013 | Measurement of arterial activity on routine FDG PET/CT images improves prediction of risk of future CV events. | No acute stroke group, measurement in aorta |
| Hjelmgren et al, 2014 | A study of plaque vascularization and inflammation using quantitative contrast-enhanced US and PET/CT. | US comparison study – no acute stroke |
| Kim et al, 2014 | Carotid inflammation on ¹⁸F-fluorodeoxyglucose positron emission tomography associates with recurrent ischemic lesions. | MRI analysis |
| Belissant et al, 2015 | Detection of a right carotid focus of 18F-FDG predicted an ischemic stroke. | Less than 5 patient analysis |
| Sharma et al, 2015 | Plaque Inflammation Imaging in Severe Carotid Stenosis and Recurrent Cerebral Ischemia. | Review of current literature |
| Emami et al, 2015 | The effect of BMS-582949, a P38 mitogen-activated protein kinase (P38 MAPK) inhibitor on arterial inflammation: a multicenter FDG-PET trial. | Effect of intervention on uptake (p38 MAPK inhibitor) |
| Kim et al, 2015 | Concurrent Carotid Inflammation in Acute Coronary Syndrome as Assessed by (18)F-FDG PET/CT: A Possible Mechanistic Link for Ischemic Stroke. | Analysis of an acute cardiac cohort |
| Buscombe et al, 2015 | Exploring the nature of atheroma and cardiovascular inflammation in vivo using positron emission tomography (PET). | Review of current literature |
| Ali et al, 2016 | FDG PET/CT Imaging of Carotid Atherosclerosis. | Review of current literature |
| Kim et al, 2016 | 18F-FDG PET/CT imaging factors that predict ischaemic stroke in cancer patients. | Analysis in a cancer cohort |
| Brinjiki et al, 2016 | Contemporary carotid imaging: from degree of stenosis to plaque vulnerability. | Review of current literature |
| Bucerius et al, 2016 | Position paper of the Cardiovascular Committee of the European Association of Nuclear Medicine (EANM) on PET imaging of atherosclerosis. | Review of current literature – position paper |
| Scherer et al, 2016 | Future imaging of atherosclerosis: molecular imaging of coronary atherosclerosis with (18)F positron emission tomography. | Review of current literature |
| Gogia et al, 2016 | Imaging High-Risk Atherosclerotic Plaques with PET. | Review of current literature |
| Kampke et al, 2017 | Diagnostic Value of Positron Emission Tomography Combined with Computed Tomography for Evaluating Critically Ill Neurological Patients. | Analysis in critically ill neurology patients |
